# Case Report: The Coronavirus Disease 2019 (COVID-19) Pneumonia With Multiple Thromboembolism

**DOI:** 10.3389/fneur.2020.625272

**Published:** 2021-01-12

**Authors:** Tingting Cao, Guqin Zhang, Huabing Xie, Emily Pellegrini, Jin Li, Xiaoxing Chen, Huaqin Pan

**Affiliations:** ^1^Department of Gastroenterology, Zhongnan Hospital of Wuhan University, Wuhan, China; ^2^Department of Respiratory and Critical Care Medicine, Zhongnan Hospital of Wuhan University, Wuhan, China; ^3^Dascena Inc., San Francisco, CA, United States; ^4^Department of Gastroenterology, The Third Affiliated Hospital of Guangzhou Medical University, Guangzhou, China; ^5^Department of Geriatrics, Renmin Hospital of Wuhan University, Wuhan University, Wuhan, China; ^6^Department of Critical Care Medicine, Zhongnan Hospital of Wuhan University, Wuhan, China; ^7^Clinical Research Center for Critical Care Medicine of Hubei Province, Wuhan, China

**Keywords:** COVID-19, SARS-CoV-2 virus, pneumonia, multiple thromboembolism, anticoagulant treatment

## Abstract

Severe acute respiratory syndrome coronavirus 2 (SARS-CoV-2) broke out in Wuhan, China, in late December 2019 and has since spread rapidly around the world. Severe coronavirus disease 2019 (COVID-19) pneumonia patients have abnormal blood coagulation function, but their thromboembolism prevalence is still unknown. We reported a case of a 49-year-old man infected with COVID-19, presenting with fever, chest pain, limb weakness, myalgia, and dyspnea. The patient was diagnosed with severe COVID-19 pneumonia, pulmonary thromboembolism (PTE), deep vein thrombosis (DVT), and cerebral infarction. He received supportive and empirical treatment including anticoagulant treatment, anti-inflammatory treatment, oxygen supply, and inhalation therapy. The patient's symptoms, CT images, and laboratory results improved after treatment, and a throat swab was reported to be negative for SARS-CoV-2 virus by polymerase chain reaction (PCR) test. However, on day 51 of illness onset, CT reexamination demonstrated hemorrhagic infarction. Anticoagulant therapy was discontinued temporarily. After the patient tested negative for SARS-CoV-2 virus by PCR test six more times, he was discharged and remained in home quarantine. This case highlights the importance of clinician attentiveness to the appearance of multiple thromboembolism, especially in patients with severe pulmonary damage. It also emphasizes the diagnostic value of early CT imaging and the need for effective treatment once thrombotic events occur.

## Introduction

The coronavirus disease 2019 (COVID-19) pneumonia is a neo-type respiratory infectious disease caused by the severe acute respiratory syndrome coronavirus 2 (SARS-CoV-2; previously known as 2019-nCoV). SARS-CoV-2 broke out in Wuhan, Hubei, China, in late December 2019 and was subsequently identified as a public health emergency of international concern by the World Health Organization (1). Although respiratory compromise with dominant symptoms of fever and cough is the cardinal feature of the disease, previous studies have reported that ~20% of COVID-19 patients have had severe coagulation abnormalities. These abnormalities predispose patients to thrombotic events such as deep vein thrombosis (DVT), venous thromboembolism (VTE), and potential pulmonary thromboembolism (PTE) and are associated with patient mortality (1–5). In this study, we report a case of a COVID-19 pneumonia patient who developed multiple thromboembolism including DVT, PTE, and cerebral infarction, which may provide further evidence for the suggestive management for such patients.

## Case

**Figure 1:**
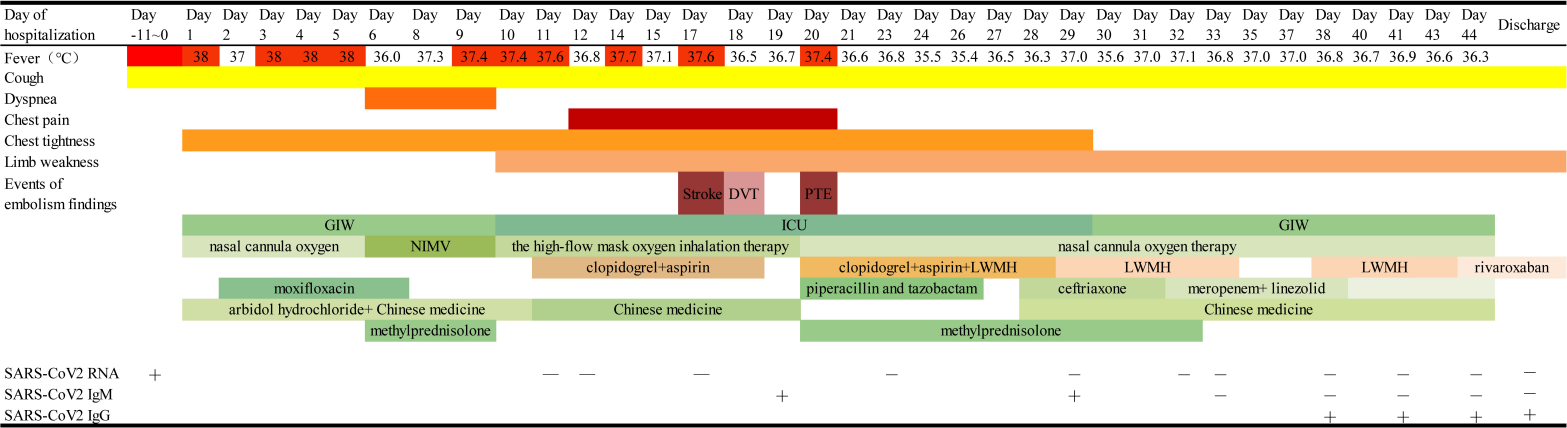
Timeline of disease course according to days from admission, and days of follow-up from February 13 to March 27, 2020. ICU, intensive care unit; GIW, general isolation ward; NIMV, non-invasive mechanical ventilation; LMWH, low-molecular-weight heparin; DVT, deep venous thrombosis; PTE: pulmonary thromboembolism.

A 49-year-old man in Wuhan sought care for a half-month history of fever up to 38°C, cough, myalgia, and dyspnea, without chills, diarrhea, nausea, vomiting, or hemoptysis. Results of a pharyngeal swab specimen analysis by the SARS-CoV-2 real-time reverse-transcriptase polymerase-chain-reaction (RT-PCR) test confirmed the patient to be positive for COVID-19 (Figure 1).

**Figure 2:**
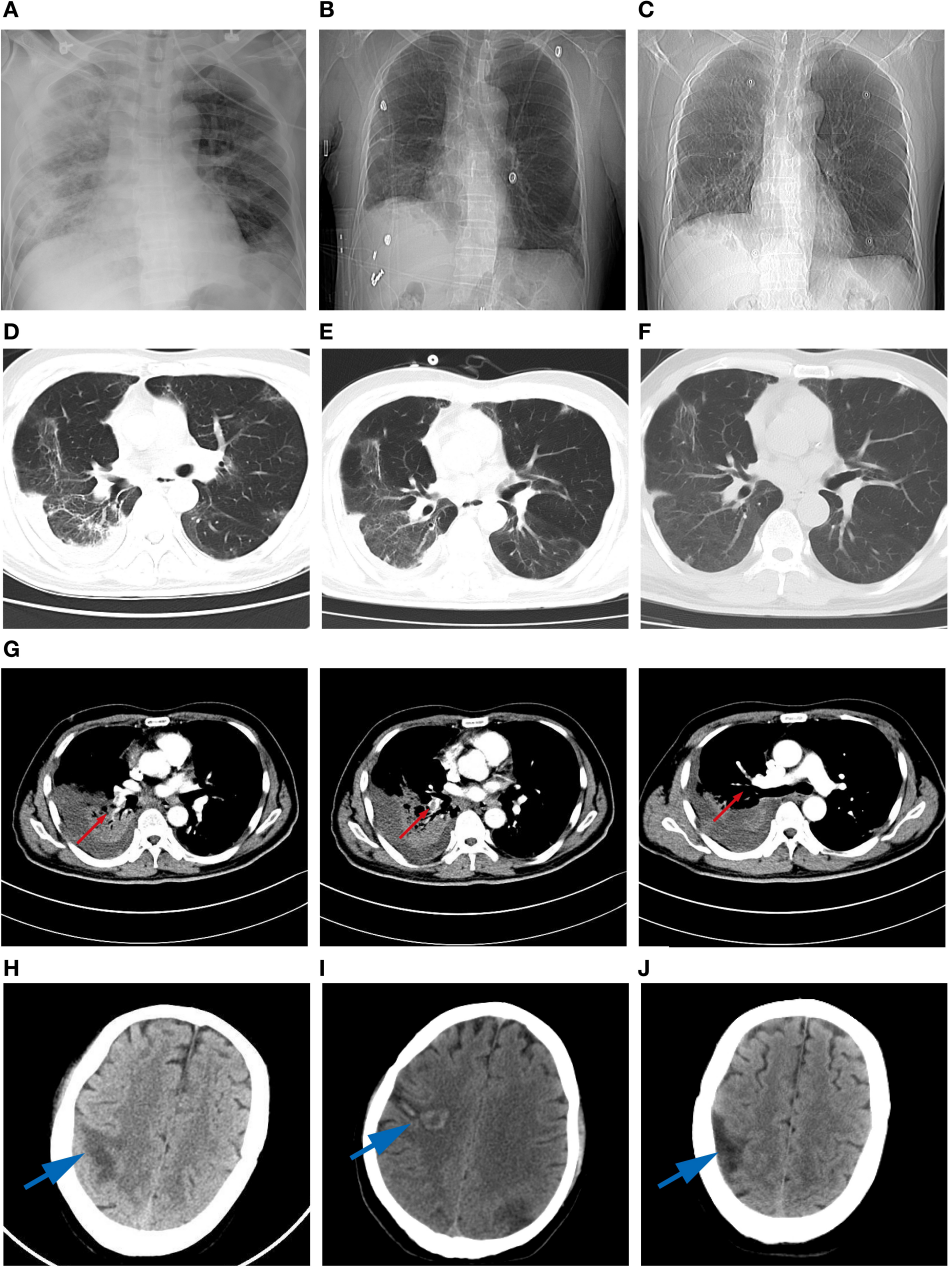
High-resolution computed tomography images during the disease course. **(A)** chest X-ray image revealing ground-glass opacities (GGO) in the both lobes on day 13 of hospitalization. **(B)** GGO in the both lobes on day 36. **(C)** Thickened lung markings in the right lobe on day 44. **(D)** Multiple-ground-glass opacification in the basal segment of lobes on day 17. **(E)** GGO in both lower lobes on day 27. **(F)** GGO in the middle lobe of the right lung on day 44. **(G)** Filling defect in the trunk and branches of the right inferior pulmonary artery (red arrow). **(H)** Slightly low-density shadows in the right parietal lobe and the left temporal lobe on day 17 of hospitalization (blue arrow). **(I)** Hemorrhagic infarction in the right parietal lobe on day 27 (blue arrow). **(J)** Low-density shadows in the right parietal lobe on day 44 (blue arrow).

On day 15 of illness onset, he was admitted to the general isolation ward (GIW) in Leishenshan Hospital and was diagnosed with severe COVID-19. Physical examination revealed a body temperature of 38.3°C, a blood pressure of 121/85 mmHg, a pulse of 102 beats per minute, and a respiratory rate of 23 breaths per minute. Laboratory results were summarized as follows:

- Lymphocyte count and percentage dramatically decreased.
- Levels of alanine aminotransferase (ALT) and aspartate aminotransferase (AST) were noticeably elevated.
- Prothrombin time prolongated, and levels of D-dimer and fibrinogen were in the normal range.
- Myocardial enzyme was in the normal range (Table 1).
- The chest computed tomography (CT) scan showed bilateral peripheral ground glass opacification (GGO) (Figure 2).

**Table 1:** Clinical laboratory results.

**Measure**	**Normal range**	**02/18 Day 6**	**02/21 Day 9**	**02/23 Day 11**	**02/24 Day 12**	**02/28 Day 16**	**03/02 Day 19**	**03/03 Day 20**	**03/03 Day 20**	**03/06 Day 23**	**03/09 Day 26**	**03/12 Day 29**	**03/14 Day 31**	**03/16 Day 33**	**03/21 Day 38**	**03/24 Day 41**	**03/27 Day 44**
WBC	3.5–9.5	7.18	8.7	11.39	9.47	7.41	6.53	6.59	6.98	5.41	7.05	3.89	4.74	3.35	3.38	2.98	3.4
PLT	125–350	99	96	84	99	298	355	366	395	439	475	378	285	242	179	155	137
NEUT#	1.8–6.3	6.91	7.95	10.37	8.35	6.14	5.45	5.16	5.88	4.16	5.29	2.37	3.42	1.98	2.24	1.7	1.61
LYMPH#	1.1–3.2	0.14	0.45	0.61	0.55	0.46	0.46	0.43	0.29	0.56	0.74	0.76	0.65	0.87	0.75	0.84	1.32
PT (S)	9.4–12.5	12.1	12	11.4	12	11.4	12.1	12.5	14.2	12.7	12.2	11.9		11.2	10.9	11.2	
APTT (S)	25.1–36.5	20.1	18.6	19.2	24.3	28.4	28.2	28.5	35.5	31.6	30.8	26.2		22.9	23.2	25.6	
DD2 (ng/mL)	0–500	28.16	30.91	21.22	12.77	6.86	5.28	4.48	3795	3973	4027	737		242	196	157	
ALT (U/L)	9–50	80	144	187	213	113	154	149	216	267	197	111		91	49	42	45
AST (U/L)	15–40	93	118	118	101	39	99	73	164	138	60	48		55	24	19	27
CREA (μmol/L)	64–104	56.9	61.5	56.3	58	46.2		56.8	45.3	50.7	57.3	67.2		61	57.1	53	61.2
CK-MB (ng/ml)	0–6.36		4.22	4.43	1.98	3.66	1.89	1.26				1.47		1.20	1.17	1.34	
HS-TNI (ng/ml)	0–0.04		0.258	0.323	0.108	0.03	0.012	0.010				0.010		0.010	0.010	0.010	
CRP (mg/L)	0–10.0				31.39	107.29	10	10	127.5	27.1		0.89		0.5	0.5	0.5	0.5
PCT (ng/ml)	<0.05	0.81	0.27	0.09	0.05	0.09	0.079		<0.05	0.05	<0.05				0.02	0.03	
IL6 (pg/mL)	0–7						88.37		87.31	9.98	12.08	147.1	215.5		120.1	81.43	
PIC (μg/mL)	<0.8								2.765	2.853	1.538	0.684		1.106	0.552		
t-PAI-C (μg/mL)	<17.0								15.6	8.8	9.3	5.9		10.0	8.6		
TM (TU/mL)	3.8–13.3								7	7.5	8.5	9.5		10.4	8.700		
TAT (ng/ml)	<4								5.2	5.3	4.1	3.3		3.4	2.9		

*WBC, white blood cell; Hgb, hemoglobin; NEUT, neutrophil; LYM, lymphocyte; ALT, alanine aminotransferase; AST, aspartate aminotransferase; CREA, serum creatinine; CRP C-reactive protein; PCT, procalcitonin; IL-6 interleukin- 6; APTT activated partial thromboplastin time; PT prothrombin time; DD D—dimer; PIC plasmin-α2-antiplasmin complex; TAT thrombin–antithrombin complex; t-PAI-C plasminogen activator inhibitor-1; TM thrombomodulin*.

The patient was supplied immediately with persistent low flow oxygen therapy, abidol hydrochloride (200 mg three times daily, po.), and Chinese medicine as antiviral therapy, and moxifloxacin (400 mg once daily, i.v.) as antibacterial therapy and symptomatic and supportive treatment. However, the patient's state became progressively aggravated despite active treatments. Five days after admission, he suddenly developed dyspnea with a lower temperature of 36.0°C, as well as a tachycardia and a decreased oxygen saturation value of 52%. He immediately received non-invasive mechanical ventilation (NIMV) because of respiratory failure. A low dose of methylprednisolone and low molecular weight heparin (LWMH) were given concurrently for prophylactic anticoagulation and anti-inflammatory based on the clinical experience. On day 7 of hospitalization, the patient's clinical condition improved and he received high-flow mask oxygen inhalation therapy (Figure 1).

On day 8 of hospitalization, the patient experienced weakness in his left upper limb and speech dysfunction. Physical examination indicated Grade 0 muscle strength in the left upper limb while other limbs were normal, the mouth was deviated, and pathological reflection of Babinski's sign was not induced; bilateral pupils were equally in diameter and sensitive to direct or indirect light reflex. Laboratory examination indicated that sensitive troponin I was 0.323 ng/ml (reference 0–0.04 ng/ml). The patient's coagulation function, CK, and CK-MB were normal. These results indicated onset of cerebral infarction. The patient was transferred to the intensive care unit (ICU).

On February 24th, the patient subsequently developed severe precordial pain. Urgent electrocardiogram indicated sinus tachycardia rather than cardiac ischemia. After treatment with nitroglycerin, atorvastatin, clopidogrel, and aspirin, the patient still had a chest pain. It was speculated that PTE was occurring. Vascular ultrasound of lower limbs showed deep vein thrombosis of the right lower extremity. Computer tomography pulmonary angiography indicated a filling defect in the trunk and branches of the right inferior pulmonary artery. Cerebral CT showed slightly low-density shadows in the right parietal lobe and the left temporal lobe (Figure 2). Laboratory reexamination indicated increases in lymphocyte count and percentage, mild decreases in AST and ALT, prolonged PT and APTT times, high platelet count, and high levels of D-dimer and fibrinogen. The patient was supplied immediately with persistent low flow oxygen therapy, low molecular heparin calcium (0.4 ml, every 12 h, im.), piperacillin and tazobactam (4.5 g, every 8 h, iv.), omeprazole (40 mg, once daily, iv.), Lipitor (20 mg, once daily, po.), aspirin (0.1 g, once daily, po), clopidogrel (75 mg, once daily, po), magnesium isoglycyrrhizinate (200 mg once daily, iv.), and a low dose of methylprednisolone (60 mg once daily, iv.).

After treatments, the patient's symptoms continued to alleviate. Laboratory reexamination indicated that the lymphocyte count and percentage increased reposefully and AST; ALT was mildly decreased while coagulation function was still not improved. Levels of D-dimer and fibrinogen remained high. The patient felt severe chest pain again on March 6. Electrocardiogram indicated sinus tachycardia. Results of a bedside chest radiograph displayed progress of bilateral interstitial infiltrating shadows. To relieve pain, the patient was given diclofenac sodium suppositories and low molecular heparin calcium continuously until day 33 of hospitalization. Amounts of methylprednisolone were gradually decreased to 20 mg on day 32 of hospitalization.

On March 11, the patient was transferred to the GIW for intensive consolidation therapy. All symptoms had resolved except for intermittent chest pain and disorder of limb activity. He received ongoing treatment in the GIW, including anticoagulant therapy with LMWH, hepatic functional protection therapy, anti-inflammatory treatment, and Chinese medicinal therapy. A CT of the lungs indicated pulmonary cavitation in the right lower lobe. Meropenem and linezolid were empirically administered as anti-infective treatments for multidrug resistance bacterial infection particularly gram-positive coccus infection. On March 17, cerebral CT indicated hemorrhagic infarction and the anticoagulant therapy was discontinued until March 20. The patient reported less pain and fatigue in his chest in the following days. He was treated with rivaroxaban (10 mg once daily, po.) when low molecular heparin calcium was discontinued. Physical examination revealed that muscle strength in the left upper limb was Grade 4; task-related dystonia was coming to normal, without numbness of the limbs, inarticulate speech, blurred vision, paresthesia, ataxia, and dystonia. Biochemistry and laboratory examination indicators gradually returned to normal levels. When the patient tested negative for SARS-CoV-2 virus by PCR test six more times, he was discharged and remained in home quarantine.

## Discussion

Coagulopathy is regarded as a common complication in patients with severe COVID-19, the clinical syndrome caused by SARS-CoV-2. The overlap in symptoms between COVID-19 and thromboembolism present a challenge for clinical diagnosis, especially for those patients without any high-risk factors. The patient we reported on in this case study was categorized as being at low-risk for venous thromboembolism according to the Padua prediction scale.

Previous studies have shown that patients with severe acute respiratory distress syndrome (SARS) have slightly decreased platelet counts and prolonged coagulation profiles and are prone to thromboembolic complications (6–9). SARS-CoV-2 shares over 88% homology with SARS coronavirus; indeed, recent studies have demonstrated that COVID-19 patients are at high risk for venous and arterial thromboembolic disease and that these diseases may be associated with increased COVID-19 severity and poor prognosis (2, 3, 10–12). Several researches described that the incidence of thrombotic complications is up to 30% of patients with COVID-19 in ICU and 2–6% of patients hospitalized with COVID-19 developed stroke (13–16). COVID-19-associated cerebrovascular manifestations seem to be mainly ischemic stroke (16–18). Most patients were generally older and with comorbidities such as hypertension, diabetes, and hyperlipoidemia, which were risk factors for ischemia stroke (18–20). Therefore, it remains ambiguous whether these strokes were caused by SARS-CoV-2 or these high-risk populations suffered strokes and also were infected at the same time. Younger patients with stroke have also been reported (9, 21). There is a study reported that widespread microthrombi and patches of infarction were observed in an autopsy series from COVID-19 patients (21). That SARS-CoV-2 infection does play some roles in causing stroke and increases stroke risk. A cross-sectional survey of 143 patients with confirmed COVID-19 pneumonia showed a 46.1% DVT incidence rate (22). The prevalence of DVT was associated with adverse outcomes, including an increased proportion of deaths, a decreased proportion of hospital discharges, and lower actuarial survival rates (22). A separate report from Cui et al. also reported that ~25% (20/81) of patients admitted to the ICU may have concurrent thromboembolic phenomena and 8 patients with VTE events died (23). While still in need of further evidence, it has been speculated that severe hypoxemia and a significant inflammatory response can lead to systemic coagulation activation (24–26). Hyperinflammation seen with “cytokines storm” and hypoxia-associated metabolic derangements are potential mechanisms of a SARS-CoV-2-related hypercoagulable state; in addition, SARS-CoV-2 may directly cause endothelial apoptosis by binding to ACE2 on endothelial cells and promote coagulation activation and thrombin generation (21, 27). This body of work suggests that adequate thromboprophylaxis and discovery in the early stages of thrombotic complications are of vital importance for the prognosis of hospitalized patients with COVID-19.

In the present case, the patient was immediately given low-molecular-weight heparin anticoagulant therapy as soon as the embolization event occurred. However, the therapeutic effect of anticoagulation on the patient was not as remarkable as was to be expected during the course of early treatment. We did not detect a decrease in fibrinogen levels and an improvement in coagulation function until heparin treatment on day 7 of hospitalization, which was slightly different from the curative effect of heparin in pulmonary embolism patients without COVID-19. Notably, the patient presented with hemorrhagic infarction during the subsequent therapy so much that it was necessary for anticoagulant therapy to be discontinued. For COVID-19 patients at high risk of bleeding and severe illness, it is therefore important that appropriate anticoagulation measures be taken to ensure effective treatment of thromboembolism. Zhai et al. recommended pharmacological prevention with low-molecular-weight heparin as a first-line treatment in patients at low or moderate risk of bleeding, as well as a curative anticoagulant with LMWH as a first-line treatment in patients suspected for VTE (28). Trigonis et al. observed that different pharmacologic prophylaxis regimens make almost no difference in the incidence of deep venous thrombosis (29). Since there is limited experience with COVID-19-associated thromboembolism, there has been no scientific consensus about the prevention and treatment for thromboembolism in COVID-19 patients.

In summary, this is a first report of a COVID-19 pneumonia patient with PTE, DVT, and cerebral infarction in Wuhan. Following an active treatment regimen consisting of anticoagulant therapy and anti-inflammatory treatment, the patient recovered well. However, due to the limited nature of this case, further research on predisposing factors and protocols for the treatment of COVID-19 pneumonia patients with multiple thromboembolisms is warranted.

## Data Availability Statement

The original contributions presented in the study are included in the article/supplementary material, further inquiries can be directed to the corresponding author/s.

## Ethics Statement

The study is approved by the Medical Ethics Committees, Zhongnan Hospital of Wuhan University (No. 2020099K), and the patient provided consent to participate. Written informed consent for publication of the clinical details and/or clinical images was obtained from the patient.

## Author Contributions

HP and TC contributed to the conceptions and design. GZ, HX, XC, and JL collected clinical data. TC wrote the raw draft. HP and EP MEng contributed to the language editing. All authors have read and approved the manuscript.

## Conflict of Interest

EP was employed by the company Dascena, Inc. The remaining authors declare that the research was conducted in the absence of any commercial or financial relationships that could be construed as a potential conflict of interest.

## Abbreviations

COVID-19: Coronavirus disease 2019
SARS-COV-2: Severe acute respiratory syndrome coronavirus 2
CT: computed tomography
ICU: intensive care unit
PTE: pulmonary thromboembolism
VTE: venous thrombus embolism
LMWH: low molecular weight heparin.
